# Immunomodulatory Effect of Ginsenoside Rb2 Against Cyclophosphamide-Induced Immunosuppression in Mice

**DOI:** 10.3389/fphar.2022.927087

**Published:** 2022-06-24

**Authors:** Siwen Zheng, Housheng Zheng, Rui Zhang, Xiangmin Piao, Junnan Hu, Yanzhu Zhu, Yingping Wang

**Affiliations:** ^1^ National and Local Joint Engineering Research Center for Ginseng Breeding and Development, Changchun, China; ^2^ College of Chinese Medicinal Materials, Jilin Agricultural University, Changchun, China; ^3^ Institute of Special Animal and Plant Sciences, Chinese Academy of Agricultural Sciences, Changchun, China; ^4^ Animal Science and Technology College, Jilin Agriculture Science and Technology University, Jilin, China

**Keywords:** ginsenoside Rb2, cyclophosphamide, side effect, immunosuppression, immune regulation

## Abstract

Ginsenoside Rb2 (Rb2), a fundamental saponin produced and isolated from ginseng (*Panax ginseng* C.A. Meyer), has a wide range of biological actions. The objective of this investigation was to see if ginsenoside Rb2 has any immunomodulatory properties against cyclophosphamide (CTX)-induced immunosuppression. For the positive control group, levamisole hydrochloride (LD) was used. We discovered that intraperitoneal injection of Rb2 (5, 10, 20 mg/kg) could relieve CTX-induced immunosuppression by enhanced immune organ index, reduced the pathological characteristics of immunosuppression, promoted natural killer (NK) cells viability, improved cell-mediated immune response, boosted the IFN-γ (Interferon-gamma), TNF-α (Tumor necrosis factor-alpha), IL-2 (Interleukin-2), and IgG (Immunoglobulin G), as well as macrophage activity like carbon clearance and phagocytic index. Rb2 significantly elevated the mRNA expression of IL-4 (Interleukin-4), SYK (Tyrosine-protein kinase-SYK), IL-2, TNF-α, and IL-6 (Interleukin-6) in the spleen of CTX-injected animals. Molecular docking results showed that Rb2 had excellent binding properties with IL-4, SYK, IL-2, TNF, and IL-6, indicating the target protein might be strongly correlated with the immunomodulatory effect of Rb2. Taken together, ginsenoside Rb2 can improve the immune function that is declined in CTX-induced immunosuppressed mice, the efficacy maybe due to the regulation of related cytokine and mRNA expression.

## Graphical Abstract

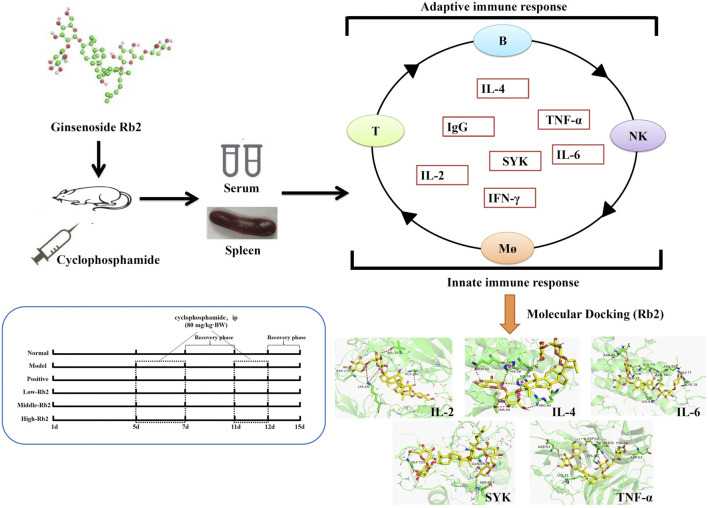

## Introduction

Cancer is internationally recognized as one of the most dangerous diseases that endanger human life and development. Chemotherapy is the main method to treat cancer. It is based on the prevention of rapid cell proliferation, which is a feature of malignant cells. Unfortunately, normal cells with quick proliferation rates, such as lymphocytes, bone marrow, and hair follicles, are also affected (Pérez-Herrero and Fernández-Medarde, 2015; Kondo et al., 2019). CTX (cyclophosphamide) is a well-known anticancer medication that is still used to treat hematological malignancies and a variety of epithelial tumors (Ahlmann and Hempel, 2016; Hughes et al., 2018). However, its treatment is often accompanied by serious side effects (Chow et al., 2017; Yang et al., 2020; Zhang et al., 2020). Immunosuppression induced by CTX is the main side effect of clinical chemotherapy. When a patient’s immune function is harmed by chemotherapy, the risk of secondary infection and immunodeficiency rises, which may result in serious morbidity and mortality problems (Yoo et al., 2020). *Lactobacillus plantarum* NCU116 was found to be capable of reversing immunosuppression caused by CTX (Xie et al., 2016), and *Paecilomyces sinensis* glycopeptides demonstrated excellent immunostimulatory capabilities (Zhen-Yuan Zhu et al., 2016).

Ginseng is one of the most widely used traditional herbal medicines, with a medical history dating back thousands of years (Ji et al., 2020). Numerous investigations into the impact of the systemic immune function of ginseng have been conducted (Riaz et al., 2019; Ratan et al., 2021). Ginsenosides are the main bioactive components in ginseng that have a variety of pharmacological actions, including the treatment of cardiovascular diseases (Kim, 2018; Irfan et al., 2020), antioxidant (Oh et al., 2015), anticancer (Wanderi et al., 2016), immune (Kang and Min, 2012; Chen et al., 2019) and osteoblast growth regulation (Yanzhu Zhu et al., 2016). Ginsenoside Rb2 (Rb2) is a highly abundant dammarane-type ginsenoside (Hong et al., 2019). Rb2 inhibits metabolic syndrome in mice such as diabetes mellitus and hyperlipidemia (Lee et al., 2011; Hong et al., 2019). Rb2 reduces the number of metastatic nodules in liver, lung and kidney of mice with colon cancer (Phi et al., 2018; Dai et al., 2019). Moreover, Rb2 protects bone marrow-derived mesenchymal stem cells from dexamethasone-induced apoptosis by increasing the GPR120-mediated Ras-ERK1/2 signaling pathway, and up-regulates GPR120 expression in lipopolysaccharide (LPS)-stimulated RAW264.7 macrophages to boost the anti-inflammatory efficacy of -3 fatty acid (Gao et al., 2015; Huang et al., 2017). Oral administration of ginsenoside Rb2 protects mice from the lethal infection of Japanese hemoagglutinating virus (Yoo et al., 2013). Thus, Rb2 is expected to be an anti-viral immune adjuvant.

The aim of this research was to explore the role of Rb2 in the immunosuppression caused by CTX. To assess the beneficial effects of ginsenoside Rb2, organ index and pathological features of spleen, splenocyte proliferation, carbon clearance, NK cell activities, mRNA expression of immune-related genes (in spleen) and cytokines (in serum) were also determined. This result will illustrate the role of Rb2 in the prevention of CTX-induced immunosuppressive effects.

## Materials and Methods

### Reagents and Animals

Jilin University (Changchun, China) provided us with ginsenoside Rb2 (purity >98%). Jiangsu Shengdi Pharmaceutical Co., Ltd. (Jiangsu China) was the source of CTX. Levamisole hydrochloride (LD) was bought from Sigma (St. Louis, MO). YAC-1 cell lines (Chinese Academy of Sciences, China) were stored by our lab. CCK-8, RPMI 1640 medium, ConA (canavalin A), LPS, Red Blood Cell Lysis Buffer, Hank’s solution, Triton X-100 and fetal bovine serum (FBS) were obtained from Beijing Solarbio Science & Technology Co., Ltd. Gibco (BRL Co. Ltd., Gaithersburg, MD, United States). IFN-γ (Interferon-gamma), TNF-α (Tumor necrosis factor-alpha), IL-2 (Interleukin-2) and IgG (Immunoglobulin G) kits were bought from Lengton Bioscience Co., Ltd. (Shanghai, China). Reverse transcription kit and qPCR kit were obtained from Trans Biotechnology Co., Ltd. (Beijing, China).

Changchun Institute of Biological Products Co. Ltd. provided one hundred and twenty male BALB/c mice (SPF level, License No. SCXK (Ji)-2017-0005). For at least a week before use, the mice were maintained in plastic cages with 12-h light/dark cycles and a relative humidity of 55% at a temperature of 23 ± 2°C. They were fed a conventional laboratory diet and had unrestricted access to water. The guiding principle was followed during the care and use of mice. The experiment was approved by the Laboratory Animal Ethics Committee of Jilin Agricultural University (Permit No. 20200923002). The experiment was repeated three times independently, and the detection of each indicator was repeated three times by different experimenters.

### Animals and Experiments Design

Six groups (*n* = 20 BALB/c mice) were prepared after a 7-day acclimation period: normal group, low-dose Rb2 administration group (5 mg/kg), middle-dose Rb2 administration group (10 mg/kg), high-dose Rb2 administration group (20 mg/kg), model group, positive group. Normal saline was given to both normal and model mice. LD (100 mg/kg) was given to the animals in the positive control group. Except for the mice in the positive group, which were administered intragastrically, the animals in other groups were administered by intraperitoneal injection. After the experiment commenced, the mice were administered for 15 consecutive days. Except for normal mice, the mice were administered with CTX (80 mg/kg) through intraperitoneal injection for three consecutive days on the 5th day and two consecutive days on the 11th day. Sixty mice (*n* = 10) were weighed individually after 24 h, while the other sixty mice were used in a carbon clearance test. The mice were euthanized by intraperitoneal injection of pentobarbital (150 mg/kg) (Alibolandi et al., 2017), and the serum was collected and centrifuged twice at 4000 rpm for 10 min. The livers and spleens were collected and weighed. The spleen indexes were computed as: index = spleen weight (g) × 1000/body weight (g). Extra spleen samples were stored in liquid nitrogen after a small quantity of spleen samples were used for tissue biopsies. Spleen samples were preserved in buffered formaldehyde at 10% (V/V), embedded in paraffin, and sectioned for histological analysis (Meng et al., 2019). 4 μm thick slices were stained with hematoxylin and eosin (HE) and captured by a microscope (OLYMPUS BX 53, Olympus Co., Tokoyo, Japan).

### Preparation of Spleen-Derived Lymphocyte Populations

After 24 h, the mice were euthanized by intraperitoneal injection of pentobarbital (150 mg/kg) (Alibolandi et al., 2017), and disinfected with ethanol for 3 min. The spleen was taken to the clean bench. Splenic cell suspension was prepared following the method (Singh et al., 2016). The connective tissue on the spleen was removed. 3 ml Hank’s was added and ground with a 10 ml syringe core. The cell suspension was screened by a 70-μm-cell-sieve, centrifugation was performed subsequently, and Red Cell Lysis Buffer was added for 3 min. Then RPMI 1640 culture medium containing 10% FBS was used to wash them twice, so as to prepare a uniform cell suspension. Spleen cell suspension was counted and checked for viability using trypan blue staining.

### Lymphocyte Proliferation

This experiment was performed as previously described (Jang et al., 2016). The CCK-8 assay was used to evaluate ConA and LPS-induced T and B splenic lymphocyte proliferation. Trypan blue staining revealed more than 95% viable cells, and the cell concentration of the cell suspension was 1 × 10^6^/ml. Con A (5 g/ml) or LPS (10 g/ml) was added to each well of 96-well plates before seeding spleen cells (100 μl). The control group consisted of spleen cells cultured in RPMI 1640 medium. The spleen cells were cultured for 48 h at 37°C in 5% CO_2_. After a 48-h incubation time, 10 μl CCK-8 chromogenic agent was supplied to each well and incubated for three additional hours. A microplate reader was used to measure the absorbance at 450 nm. The detection was repeated three times by different experimenters.

### Determination of Natural Killer Cell Activity

Lactate dehydrogenase (LDH) release test was used to measure NK cell activity (Hwang et al., 2022). NK cells were employed to treat pre-cultured YAC-1 cells. The concentration of target cells was adjusted to 4 × 10^5^ cells per milliliter. Splenocytes from mice’s spleens were used as a source of effector cells, and the cell concentration was controlled to 2 × 10^7^ cells per milliliter using RPMI 1640 media containing 10% FBS.

Effector cells were co-cultured with target cells (100 μl) at a 50:1 ratio in an U-shaped 96-well plate. For the experiment, two controls were used: a spontaneous control and a maximum spontaneous control. As a spontaneous control, target cells (100 μl) and RPMI1640 medium (100 μl) were added to the natural target cells release well. A maximal spontaneous control was created by adding target cells (100 μl) and Triton X-100 (100 μl, 2.5%, v/v) to the maximum releasing well. Plates were incubated at 37°C for 4 h in a 5% CO2 incubator before being centrifuged for 5 min at 900 g. Supernatant (100 μl) was transferred to a new plate, followed by 100 μl of LDH matrix solution and 30 μl of 1 M HCL in each well. Finally, at 490 nm, the absorbance value was determined by an automatic microplate reader (BioTek Epoch, BioTek, Winooski, VT, United States). The detection was repeated three times by different experimenters.

### Carbon Clearance Test

Chen’s approach was used to conduct the carbon clearance test (Chen et al., 2019). Each mouse (six groups, *n* = 10) received an intravenous injection of 4 times diluted India ink at a dose of 100 μl/10 g. After 2 min (t1) and 10 min (t2), 20 μl blood was collected from the retinal venous plexuses and mixed with 2 ml of 0.1% Na_2_CO_3_ at once. The absorbance was measured at 600 nm in an ELISA reader. Mice were euthanized by cervical dislocation (Karimi et al., 2018), and their spleen and liver were removed and weighed. Phagocytic index α was used to express the carbon clearance ability of the mice. The detection was repeated three times by different experimenters.

### Measurement of Immune-Related Cytokines in the Serum

The serum was obtained before the animals were euthanized (ip., Pentobarbital, 150 mg/kg) and centrifuged twice at 4000 rpm for 10 min. The manufacturer’s instructions were followed to assess the levels of IL-2, TNF-α, IFN-γ, and IgG in the serum using mouse enzyme-linked immunosorbent assay kit. The detection was repeated three times by different experimenters.

### qRT-PCR Analysis

The reverse transcription method was based on the instructions of the kit. The dose of reactants was adjusted according to the concentration of total RNA extracted from tissues. After successful reverse transcription, samples were stored at −80°C. The functional gene expression was determined by the TransStart Top Green qPCR SuperMix (+dye Ⅱ) method in QuantStudio® three real-time quantitative PCR system. 2-ΔΔCT approach was used to obtain the mRNA relative expression. The primer sequences of the target gene and housekeeper gene determined in this study are shown in Table 1. The detection was repeated three times by different experimenters.

**TABLE 1:** Primer sequences (5′–3′) used for qRT-PCR.

Gene	Forward sequence	Reverse sequence
IL-2	TAC​AGC​GGA​AGC​ACA​GCA​G	CGC​AGA​GGT​CCA​AGT​TCA​TC
IL-4	AAC​GAG​GTC​ACA​GGA​GAA​GG	TGG​AAG​CCC​TAC​AGA​CAA​GC
IL-6	CGG​AGA​GGA​GAC​TTC​ACA​GAG	CAT​TTC​CAC​GAT​TTC​CCA​GA
SYK	CAG​CTG​GAG​GAT​CGG​AGA​AC	CCA​TGG​AAC​CAG​GGC​ATC​TT

### Molecular Docking

In order to explore the binding site and binding force between Rb2 and IL2, IL-4, IL-6, Syk, and TNF, we used molecular docking technology. The steps of molecular docking were as follows: download the protein structure and preprocess, download the ligand structure and preprocess, conduct molecular docking, calculate the binding score, and visualize the docking results (Ferdian et al., 2020). Canonical SMILES notations of Rb2 were retrieved from PubChem Databases in NCBI (National Library of Medicine) and PDB structure files was created by ChemOffice 2017. Protein structures for SYK (PDB ID: 2mc1), TNF (PDB ID: 2tnf), IL-2 (PDB ID: 4YUE), IL-4 (PDB ID: 2B8U), and IL-6 (PDB ID: 2I3Y) were available from the Protein Data Bank. The Lamarckian genetic process was used to convert the format and find the active pocket with AutoDockTools-1.5.6 (Molecular Graphics Laboratory, The Scripps Research Institute, La Jolla, CA, United States). The pretreated protein ligands and protein receptors were docked with Autodock-Vina (Molecular Graphics Laboratory, The Scripps Research Institute, La Jolla, CA, United States) for three times independently. Docking log files yielded the lowest binding energy. The mean of binding energy was calculated from three independent dockings. Selected dockings were visualized with PyMoL software (Schrödinger, LLC, New York, NY, United States).

### Statistical Analysis

Experimental data were analyzed utilizing Graphpad Prism 7.0 (GraphPad Software Inc., La Jolla, CA, United States). The data were presented as means ± S.D. One-way ANOVA with *t*-tests for post hoc analysis was used to assess the differences between the groups. If the difference was less than 0.05, it was regarded as significant, and if it was less than 0.01, it was considered extremely significant. ##*p* < 0.01, compared to Normal. *p < 0.05; **p < 0.01, compared to Model.

## Results

### Body Weight and Spleen Index

As indicated in Table 2, CTX-induced immunosuppression resulted in significant weight loss of the body and spleen indexes. However, the mice with different doses of Rb2 showed significantly larger spleen indices than the CTX group (*p* < 0.05). When compared to the CTX group, the body weight of the positive control and middle-Rb2 groups improved significantly (*p* < 0.05). It indicates that ginsenoside Rb2 restored the spleen indices and the body weight in the mice with CTX.

**TABLE 2:** Effect of ginsenoside Rb2 on body weight and spleen indices in mice (mean ± S.D., *n* = 10).

Group	Dose (mg/kg)	Initial body weight (g)	Final body weight (g)	Spleen index (mg/g)
Normal	-	26.77 ± 1.64	28.95 ± 1.16	5.53 ± 0.91
Model	-	27.00 ± 1.67	23.81 ± 2.66^##^	2.14 ± 0.31^##^
Positive	100 (LD)	27.92 ± 1.10	26.89 ± 1.50^*^	3.46 ± 0.89^**^
Low-Rb2	5	26.58 ± 1.57	23.99 ± 2.20	3.00 ± 0.89^*^
Middle-Rb2	10	26.84 ± 1.93	26.92 ± 1.49^*^	3.57 ± 0.66^**^
High-Rb2	20	27.38 ± 1.98	26.12 ± 2.43	3.86 ± 0.50^**^

### Histopathological Alterations

**FIGURE 1:**
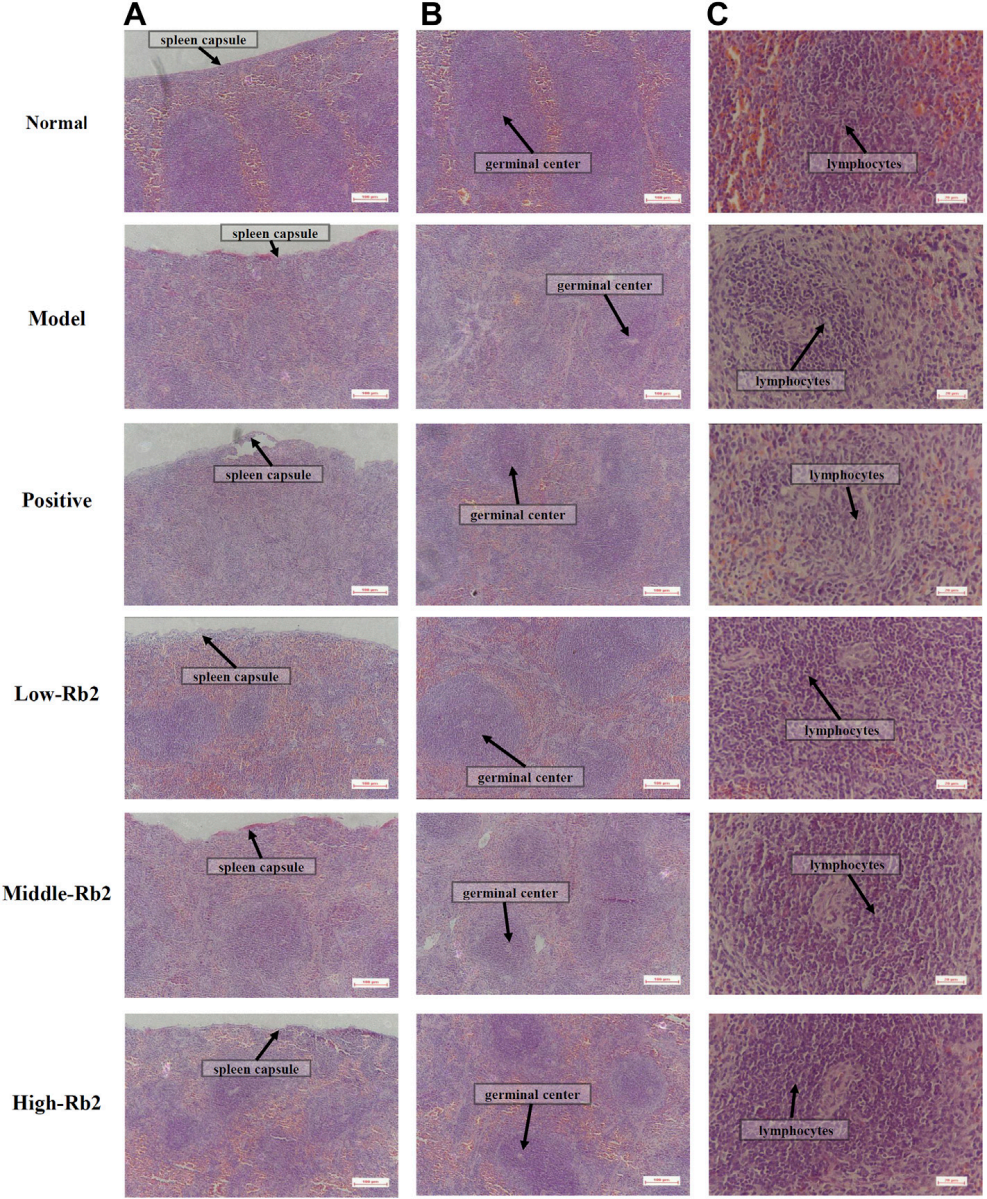
Histopathology observation of spleen. **(A)** ×100, **(B)** ×100, **(C)** ×400.

The spleen histopathology was examined with H&E staining to further analyze the effect of Rb2. The spleen capsule was damaged, the germinal center was distributed, and the number of lymphocytes was lowered and sparse in the model group (Figure 1). The damage to the spleen was relieved to varying degrees after Rb2 treatment compared to the model, the spleen capsule was intact, and the germinal center was integrated. The lymphocytes had increased considerably, and they were tightly packed together. The findings showed that Rb2 reduces CTX-induced spleen damage in mice.

### Splenic Lymphocytes Proliferation

**FIGURE 2:**
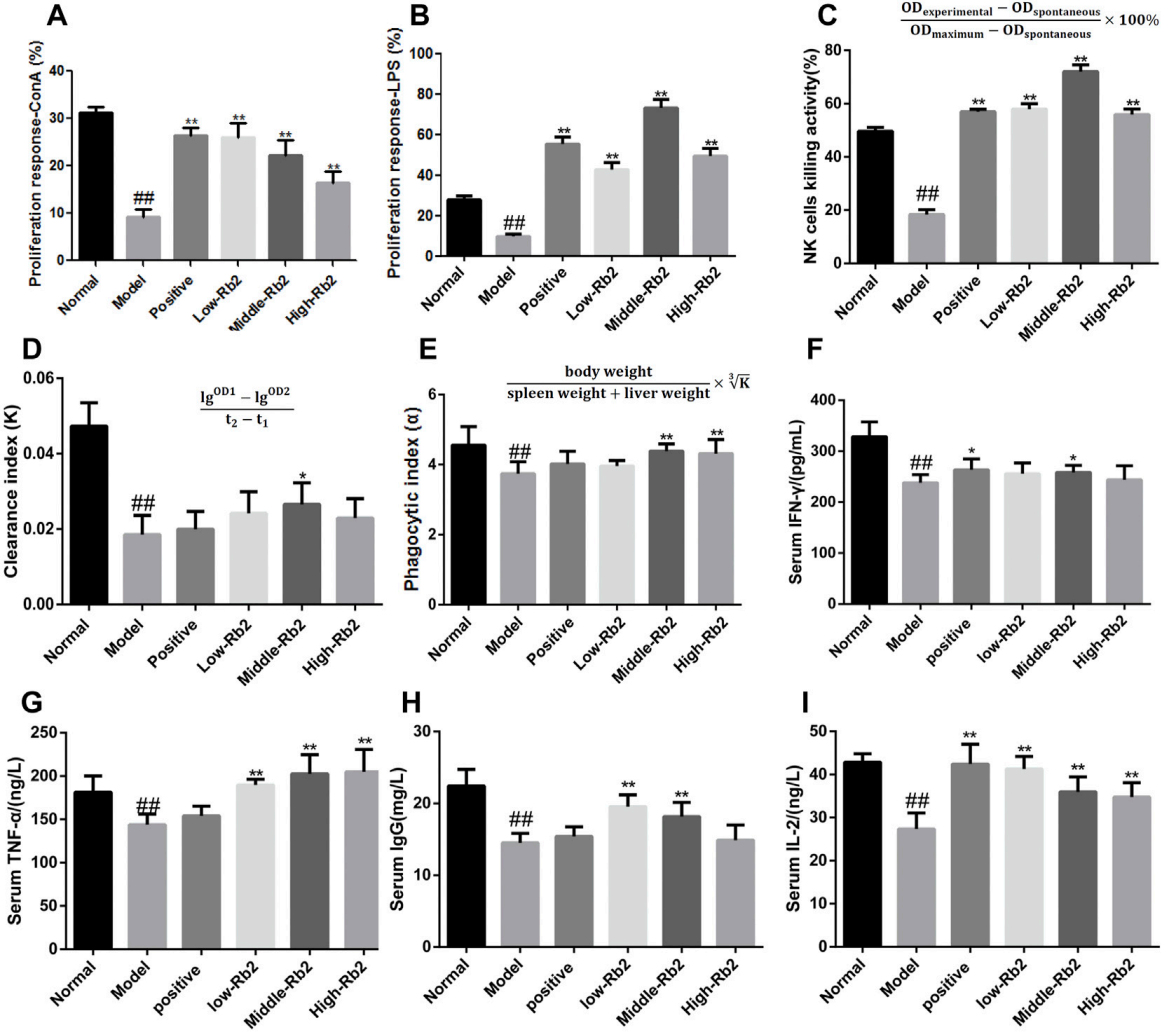
Effect of ginsenoside Rb2 on ConA- and LPS- induced splenocyte proliferation, on NK cells activity, carbon clearance capacityin and the cytokines in CTX induced immunosuppressive mice. **(A)**, ConA-induced splenocyte proliferation; **(B)**, LPS-induced splenocyte proliferation; **(C)**, NK cells activity; **(D,E)**, carbon clearance capacityin; **(F)**, IFN-γ; **(G)**, TNF-α; **(H)**, IgG; **(I)**, IL-2. The data was expressed as mean ± S. D (*n* = 20). ##*p* < 0.01, compared to Normal. *p < 0.05; **p < 0.01, compared to Model.

T and B lymphocyte proliferation in response to mitogens or antigens has traditionally been used as an immunological measure to assess lymphocyte responsiveness. ConA-induced cellular proliferation was used to assess T lymphocyte immunity, while LPS-induced B cell activation was used to identify B lymphocyte immunity. In the model group, the proliferation index of splenic lymphocytes to ConA (Figure 2A) and LPS (Figure 2B) was considerably lower than the normal control group (*p* < 0.05). The proliferation index of splenic lymphocytes in immunosuppressed mice was considerably increased after treatment with Rb2 compared to the model group (*p* < 0.05). Rb2 was found to be involved in the splenic T and B lymphocytes proliferation in immunosuppressed mice induced by CTX.

### Natural Killer Cell Activity

LDH release test was used to assess NK cell activity. Figure 2C demonstrates that the model group’s NK cell activity was dramatically reduced to 18% (*p* < 0.01) compared to the normal control group. Compared to the model group, the activity of NK cells in the positive group, the low-Rb2 group, the middle-Rb2 group and the high-Rb2 group was significantly increased to 57, 58, 72, and 56%, respectively. It indicates that Rb2 boosts cell immunological function in mice with CTX.

### Effects of Ginsenoside Rb2 on Carbon Clearance

To investigate the influence of Rb2 on macrophage activation, carbon clearance assays were performed. The CTX-treated group had a considerably lower phagocytic index α than the normal control group (*p* < 0.01) (Figures 2D,E). Pretreatment with Rb2 dramatically reversed the inhibitory effect of CTX. The values in the middle-Rb2 (*p* < 0.05) and high-Rb2 groups (*p* < 0.01) were considerably higher than those in the model group, suggesting that Rb2 can improve macrophage function in immunosuppressed mice caused by CTX.

### Cytokines

To explore the immunomodulatory effects of Rb2 on cytokines, IFN-γ, TNF-α, IL-2, and IgG levels in serum were assessed. As shown in Figures 2F–I, the levels of immune-related cytokines in the model were considerably lower (*p* < 0.01) than the control. The IFN-γ content in the positive and middle-Rb2 group was substantially higher compared to the model (*p* < 0.05), whereas that in the low-Rb2 and high-Rb2 groups exerted an increased trend. The content of TNF-α and IL-2 in the three Rb2 administration groups was significantly enhanced (*p* < 0.01). IgG content in the low and middle groups was enormously improved (*p* < 0.01), but IgG was not significantly regulated in high group.

### The Levels of Immune-Related Gene Expression

**FIGURE 3:**
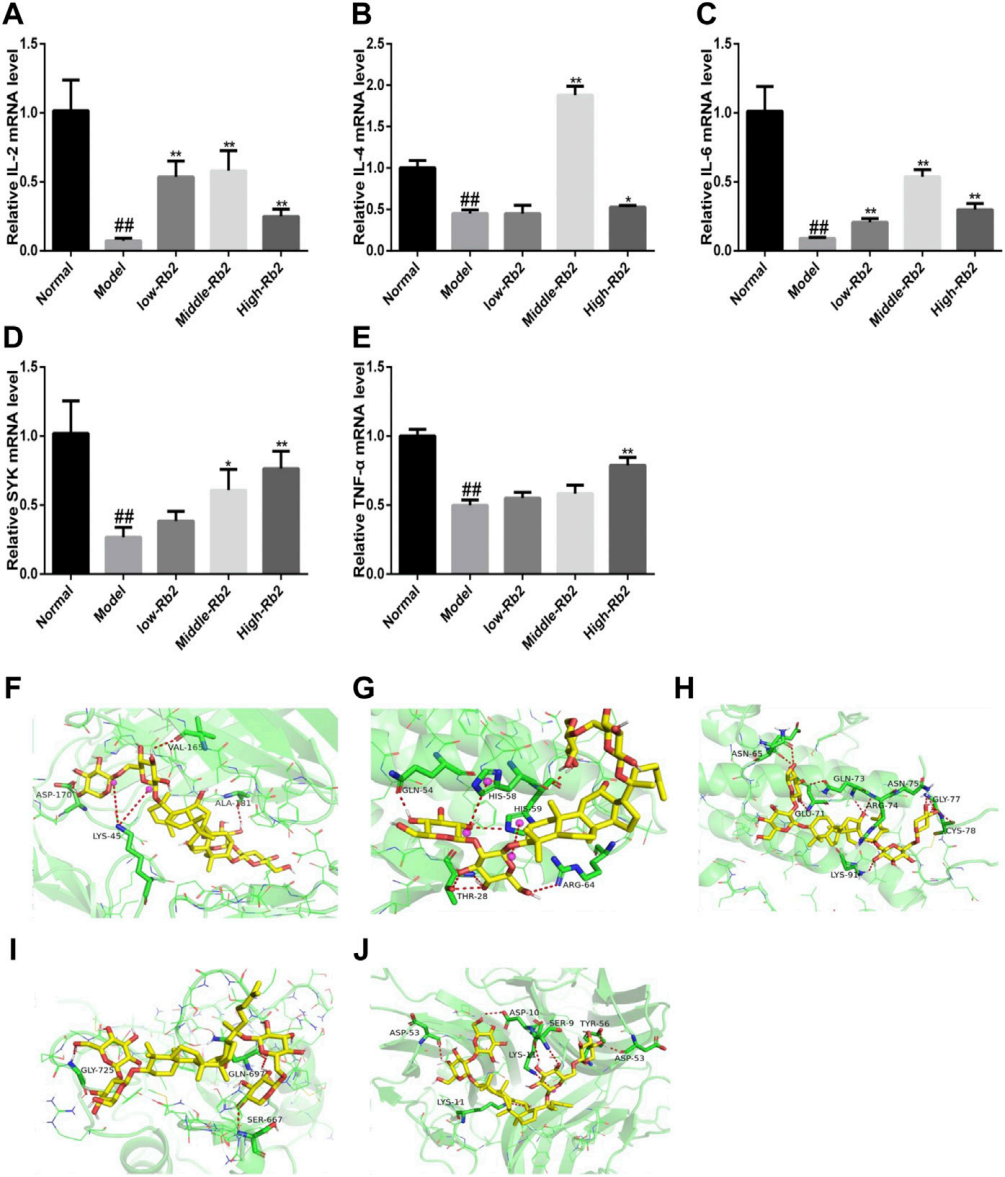
The relative gene expression of cytokines in spleen. **(A)**, IL-2; **(B)**, IL-4; **(C)**, IL-6; **(D)**, SYK; **(E)**, TNF-α. ##*p* < 0.01, compared to Normal. **p < 0.01, compared to Model. **(F)** Docking of Rb2 (yellow) into the binding site of mouse IL-2 (PDB code: 4yue, green); **(G)** Docking of Rb2 (yellow) into the binding site of mouse IL-4 (PDB code: 2b8u, green); **(H)** Docking of Rb2 (yellow) into the binding site of mouse IL-6 (PDB code: 2l3y, green); **(I)** Docking of Rb2 (yellow) into the binding site of mouse SYK (PDB code: 2mc1, green); **(J)** Docking of Rb2 (yellow) into the binding site of mouse TNF (PDB code: 2tnf, green).

Compared with the control, CTX significantly reduced the above-mentioned relative gene expression levels (Figures 3A–E) in the model (*p* < 0.01). The relative expression of IL-4, TNF-α, IL-2, SYK, and IL-6 was promoted after treatment with Rb2.

### Docking Results

In order to investigate the binding pattern and the active site cavity of Rb2 and IL-4, SYK, IL-2, TNF, and IL-6, molecular docking was accomplished by AutoDockTools-1.5.6 and AutoDock vina. The lowest binding energies for TNF, IL-4, SYK, IL-2, and IL-6 were −7.7, −7.3, −7.6, −9.8 and −8.3 kcal/mol, respectively. The result indicates that Rb2 has a higher affinity for IL-2 than the other proteins. Molecular docking investigations of Rb2 on IL-2 (Figure 3F) revealed hydrogen bonding interactions as well as a salt bridge with the amino acid residues around the binding site. Rb2 binds with IL-2 by forming three hydrogen bonds as illustrated in Figure 3F (amino acid residues ASP-170, VAL-165 and ALA-180), and the salt bridge was evident with amino acid residues LYS-45 (2 bonds). Hydrogen bonds between Rb2 and IL-4 were formed with amino acid residues GLN-54, THR-28 (3 bonds) and ARG-64, and the salt bridge was evident with amino acid residues HIS-58 and HIS-59. The binding interactions of Rb2 on IL-6 have been shown in the form of hydrogen bonds in molecular docking studies. The amino acid residues ASN-65 (2 bonds), GLU-71, GLN-73 (2 bonds), ARG-74, LYS-91, ASN-75 (2 bonds), GLY-77 (2 bonds), and CYS-78 were identified as forming hydrogen bonds with Rb2 (Figure 3H). Rb2 binds to SYK via hydrogen bonds. The amino acid residues GLY-725, GLN-697, and SER-667 (Figure 3I) were all discovered to form hydrogen bonds with Rb2. Additionally, Rb2 was found to interact with TNF by forming hydrogen bonds with amino acid residues ASP10, SER-9 (2 bonds), TYP-56, ASP-53 (2 bonds), and LSP-11, and the salt bridge was evident with amino acid residues LYS-11 (Figure 3J).

## Discussion

Almost all chemotherapeutic medicines are toxic, and chemotherapy has substantial and often fatal adverse effects, such as severe nausea, myelosuppression, and immunosuppression (Ngulde et al., 2020). CTX is an immunosuppressant that is frequently used in clinical chemotherapy for malignant tumors. The proliferation of T and B was inhibited by CTX through disrupting with DNA and RNA activities (Johnson et al., 2012). Ginseng is well known for its immune-boosting properties. The modulation of the immune system has been extensively described in terms of preserving immune system homeostasis and increasing resistance to disease or pathogenic attacks (Lee et al., 2015; Iqbal and Rhee, 2020).

Ginsenosides (Re, Rg3, Rg1, Rb1, Rb2) are the major bioactive constituents of ginseng, which dramatically amplify immune function in mice (Song et al., 2010; Park et al., 2011; Wang et al., 2014). Combining CTX with ginsenosides can improve the anti-cancer activity of CTX. The enhancing effects were responsible for the increase in anti-cancer immunity (Chen et al., 2017; Zhu et al., 2021). The immune system, which consists of innate and adaptive immunity, is the human’s ultimate defense against infectious illnesses, tumor and cancer growth. In this investigation, we used a variety of assays to assess the effects of Rb2 on immune responses. The tests showed that Rb2 improved cellular, macrophage phagocytosis and NK cell activity in animals that had been given CTX, reducing the negative effects of immune suppression. The immunomodulatory effect of Rb2 is due, at least in part, to the related cytokine regulation and mRNA expression.

Spleen indices can reveal information about an organism’s immunological activity and prognosis. In this study, the spleen weight was increased in the Rb2 groups compared to the model and control group. The adaptive immune response’s key cell component is the lymphocyte. T and B lymphocyte proliferation in response to mitogen or antigen is a common immunological measure used to evaluate lymphocyte response. In general, T lymphocyte immunity can be detected using ConA-induced cell proliferation, whereas B lymphocyte immunity may be detected using LPS-induced B cell activation (Monmai et al., 2017; Liu et al., 2018). It indicated that ginsenoside Rb2 reversed the effect of CTX on splenic T and B lymphocyte proliferation.

Macrophages and NK cells play an important role in the anti-microbial response (Bihl et al., 2011; Orange, 2013; Shapouri-Moghaddam et al., 2018). Macrophages regulate innate immune protection against pathogens, as well as adaptive immunological responses. Macrophages are well-known for being highly secretory cells. Macrophages coordinate the immune response by secreting a variety of cytokines in response to stimulation. In numerous experiments, ginseng extracts were found to increase macrophage phagocytic activity. In the innate immune system, NK cells are a type of cytotoxic lymphocyte that fights freshly formed malignant cells and infected cells. After being stimulated, macrophages begin phagocytosis and produce a variety of effector molecules, including NO, to protect the host from damage (Kovacevic et al., 2017). NK cells participate in immune surveillance by destroying non-specific target cells and releasing IFN-γ. Further, NK cells activate macrophages to eliminate phagocytized microorganisms (O'Sullivan et al., 2015). Thus, macrophages and NK cells are contributing to tumor monitoring and pathogen clearance (Cerwenka and Lanier, 2016). In our experiment, Rb2 can improve macrophage and NK cell function, indicating that it could improve cell immunological function.

Innate and acquired immunity against microbial invasion, as well as the formation and activation of effector cells, also rely on cytokines. They are tiny signaling molecules produced by a variety of cells that help to mediate immune responses. Innate immunity is regulated by IL-10, TNFs, IL-12, and IL-6, which are produced by macrophages and NK cells. IFN-γ, IL-2, TNF-β, IL-12 were secreted by Th1 cells, and IL-10, IL-4, IL-13, and IL-5 were secreted by Th2 cells. Th1 and Th2 cells differ in the cytokines they secrete, which determines their differentiation and biological roles. IL-2 is a major immune factor secreted by Th cells. Combined with IL-2R, IL-2 can stimulate immune cell proliferation while inhibiting malignant cell division (Wang et al., 2005; Sockolosky et al., 2018). Moreover, IL-2 enhances the activity of NK cells like interferon. Thus, IFN-γ production is increased (Ferlazzo et al., 2004; Wang et al., 2012). IL-2 can also promote B cell differentiation and IgG secretion from naive B cells stimulated by IL-21 (Berglund et al., 2013). IFN-γ is an effective activator of macrophages. After CTX exposure, the Rb2 treated groups had significantly improved NK cell activity, which could be associated with greater amounts of IL-2. According to the findings, Rb2 reversed the significant depression caused by CTX by increasing the levels of IFN-γ, TNF-α, IL-2, and IgG.

IL-4, IL-2, and IL-6 have been shown in numerous studies to stimulate immune cell activity and boost overall immunity. In immunosuppressed mice, Rb2 was observed to boost the mRNA expression of IL-2, SYK, IL-4, TNF-α, and IL-6. SYK is a protein tyrosine kinase that is found in hematopoietic cells in large amounts (Toyabe et al., 2010). They activated immunological receptors and downstream signaling to control cell proliferation, differentiation and phagocytosis (Siegel et al., 2006). Molecular docking is a popular computational approach for researching intermolecular interactions between molecular. From molecular docking study, it was found that IL-4, IL-2, SYK, IL-6, and TNF exhibited enough favorable interactions with Rb2. Hence, the results confirmed that Rb2 can enhance immune activity through regulating the production of IL-2, SYK, IL-4, TNF, and IL-6 in CTX-induced immunosuppressed mice.

## Conclusion

The current findings showed that ginsenoside Rb2 reversed CTX-induced spleen damage, increased splenic T and B lymphocyte proliferation, improved macrophage function and NK cell activity, and increased cytokine production and mRNA expression. It suggests that ginsenoside Rb2 reversed CTX-induced immunosuppression in the mice.

## Data Availability

The original contributions presented in the study are included in the article/supplementary materials, further inquiries can be directed to the corresponding authors.
